# Shared genomic segment analysis with equivalence testing

**DOI:** 10.1002/gepi.22335

**Published:** 2020-07-16

**Authors:** Sukanya Horpaopan, Cathy S. J. Fann, Mark Lathrop, Jurg Ott

**Affiliations:** ^1^ Department of Anatomy, Faculty of Medical Science Naresuan University Phitsanulok Thailand; ^2^ Department of Biomedical Sciences, Institute of Biomedical Sciences Academia Sinica Taipei Taiwan; ^3^ Department of Genome Quebec Innovation McGill University and Genome Québec Innovation Centre Montréal Québec Canada; ^4^ Department of Statistical Genetics, Laboratory of Statistical Genetics Rockefeller University New York New York

**Keywords:** ALSPAC, computer simulation, equivalence testing, gene mapping, genetic association analysis, sequence variants

## Abstract

An important aspect of disease gene mapping is replication, that is, a putative finding in one group of individuals is confirmed in another set of individuals. As it can happen by chance that individuals share an estimated disease position, we developed a statistical approach to determine the *p*‐value for multiple individuals or families to share a possibly small number of candidate susceptibility variants. Here, we focus on candidate variants for dominant traits that have been obtained by our previously developed heterozygosity analysis, and we are testing the sharing of candidate variants obtained for different individuals. Our approach allows for multiple pathogenic variants in a gene to contribute to disease, and for estimated disease variant positions to be imprecise. Statistically, the method developed here falls into the category of equivalence testing, where the classical null and alternative hypotheses of homogeneity and heterogeneity are reversed. The null hypothesis situation is created by permuting genomic locations of variants for one individual after another. We applied our methodology to the ALSPAC data set of 1,927 whole‐genome sequenced individuals, where some individuals carry a pathogenic variant for the BRCA1 gene, but no two individuals carry the same variant. Our shared genomic segment analysis found significant evidence for BRCA1 pathogenic variants within ±5 kb of a given DNA variant.

## 1 INTRODUCTION

Shared genomic segment (SGS) analysis refers to detecting short segments of DNA shared by two or more individuals more than expected by chance. The aim has often been to estimate relatedness among individuals or to find genes underlying a trait shared by these individuals. In one of the earliest of such approaches, a rare autosomal recessive trait was mapped to a segment on chromosome 18 on the basis of four affected individuals (Houwen et al., 1994). More recently, an SGS approach was developed for use in extended family pedigrees (Thomas, Camp, Farnham, Allen‐Brady, & Cannon‐Albright, 2008). The aim was to find long runs of loci at which affected individuals share a common allele in the expectation that such runs harbor disease susceptibility loci. For statistical significance, a long run in a candidate region was compared with run lengths in the rest of the genome. This approach was recently applied to multigenerational pedigrees with gastroschisis (Feldkamp et al., 2019).

In a somewhat different approach, long runs of heterozygous variants (single nucleotide polymorphisms, SNPs) were compared with expected lengths of such runs, where distributions of expected run lengths were obtained by computer simulation (Dimitromanolakis, Paterson, & Sun, 2019). Analogous calculations were carried for markers homozygous for the wild‐type allele. The sharing of long runs of markers is then carried out for all pairs of individuals.

Here, we focus on individuals affected by an inherited dominant trait and use our previously developed heterozygosity analysis (HA; Imai‐Okazaki et al., 2019) to generate a relatively small number of candidate DNA variants for each individual. Then we develop an approach to assess statistical significance for the sharing of such candidate variants among affected individuals as follows. The traditional approach to disease gene mapping, for example, by linkage analysis, has been to initially assume homogeneity among families (null hypothesis, H_0_: linkage with homogeneity) and search for high lod scores (Morton, 1955). A subsequent analysis then tests for heterogeneity (alternative hypothesis, H1: linkage with heterogeneity) to see whether potential disease loci occur at different genomic locations in different families (Morton, 1956). This concept is rather counterintuitive for highly heterogeneous traits such as psoriasis (Rendon & Schakel, 2019) and Charcot–Marie–Tooth disease (Bird et al., 1983; Morena, Gupta, & Hoyle, 2019), in which it is more natural to expect that high lod scores would occur at multiple places in the genome, and their occurrence in two or more families at the same approximate genomic location is unexpected. Thus, we propose an approach that reverses the traditional hypothesis testing scenario. Assume that for each individual or group of individuals like a family, a number of candidate susceptibility variants have been obtained. We initially assume that these variants can occur anywhere in the genome (null hypothesis, H0: heterogeneity). Under this null hypothesis, the fact that variants in multiple individuals occur at approximately the same position is an unlikely occurrence; in fact, two such variants have an approximate probability of only 5% to even occur on the same chromosome (Smith, 1953), and a much smaller probability to occur within, say, 50 kb of each other. This “surprise factor” has previously been expressed in an *ad hoc* manner (Rodelsperger et al., 2011; Sherman et al., 2008) but here we develop a testing framework, where homogeneity is our *alternative* hypothesis (H1).

This reversal of null and alternative hypotheses is known as *equivalence testing*, where the name refers to the fact that in testing the efficacy of a new drug it is often desired to show that it is equal (equivalent) to an existing drug. In human genetics, equivalence testing has rarely been applied (Lin, 2002; Lin, Rogers, & Hsu, 2001; Wellek & Schumann, 2004), but it has recently seen a resurgence of interest (Lakens, 2017; Lakens, McLatchie, Isager, Scheel, & Dienes, 2020). In our approach, we assume a number of individuals affected with a genetic trait, each being genotyped for a large number of DNA variants. For each given individual, a suitable test statistic generally identifies multiple candidate disease variants and we are interested in finding disease variants that are significantly shared by several individuals.

## 2 ESTIMATING POSITIONS OF CANDIDATE DOMINANT DISEASE VARIANTS

In our approach, we focus on dominantly inherited disease variants and estimate their positions with a recently developed method, HA (Imai‐Okazaki et al., 2019), but the approach developed here is rather general. Briefly, DNA variants in the vicinity of a rare dominant trait variant tend to show a heterozygote excess (Klein et al., 1998). In fact, if *f* is the minor population allele frequency of a variant in the immediate vicinity of an inherited dominant trait variant (population heterozygosity = 2 *f*[1 – *f*]), then variants in the immediate vicinity of the dominant trait variant tend to be heterozygous with probability P(*H*) > 1 – *f* (Imai‐Okazaki et al., 2019). Thus, the discrepancy in heterozygosity for variants far from versus close to a dominant trait variant is particularly striking for small *f*.

To accommodate variants with varying heterozygosity, we work with a “window” of (2*m* + 1) adjacent variants, that is, we consider a given DNA variant and the *m* variants on either side of it. Average heterozygosity, *H*, is then computed as *h*/(*g* + *h*), where *g* and *h* are numbers of homozygous and heterozygous genotypes at the (2*m* + 1) variants, respectively. A suitable value is *m* = 50, but our implementation of HA dynamically determines optimal values of *m* (Imai‐Okazaki et al., 2019; *PH* program, http://lab.rockefeller.edu/ott/programs). We slide such a window of variants, one variant at a time, from one end to the other of each chromosome. Thus, except for *m* variants at chromosome ends, there are as many *H* values as DNA variants.

As inherited dominant disease variants tend to be surrounded by DNA variants with increased heterozygosity, the next step is to determine, for each individual, runs of increasing heterozygosity up to a maximum, *H*_max_, followed by variants with decreasing heterozygosity. While the number of *H* values can be several million, only a few dozen of such runs and associated *H*_max_ values tend to emerge and known disease variants are generally found close to one of the *H*_max_ positions. Details are provided in our previous publication (Imai‐Okazaki et al., 2019). As outlined in the next section, here we only work with *H*_max_ values (however many occur for an individual) and consider their positions as candidate disease variant locations for that individual.

## 3 SHARING GENOMIC SEGMENTS

Consider a number *N* of individuals affected with a dominantly inherited trait. We are interested in finding candidate susceptibility variants for this trait. Specifically, we want to find susceptibility variants that are shared among a number *N*_f_ < *N* of individuals, where the random probability of such sharing is small, *p* < .05. We start out by creating, for each of the *N* individuals, a set of positions, *k*, of *H*_max_ values, which will be done with our *PH* program implementing the HA algorithm. In our experience, the number *k* of *H*_max_ values per individual tends to vary between 10 and 200, depending on the data analyzed.

For each DNA variant in the genome, we determine for each individual whether any of their *k* estimated disease variant positions fall within a distance ±*d* kb from the position of the DNA variant. If this happens for two or more individuals, then these individuals are said to share that segment of width 2*d* surrounding the given DNA variant. We carry out such a determination for each DNA variant, which results in a number *N*_f_ of individuals sharing a given variant, where *N*_f_ is our test statistic. To determine the random probability of sharing, we assume as the null hypothesis that a given variant position for a given individual can be anywhere in the genome. Thus, for each individual separately, we randomly permute positions of DNA variants and carry out the above procedure for these pseudo‐positions of DNA variants, with each permutation resulting in a pseudo‐*N*_f_ value. The *p*‐value associated with an observed number *N*_f_ is then estimated by the proportion of pseudo‐*N*_f_ values greater than or equal to the observed *N*_f_ value.

## 4 PROOF OF CONCEPT: POPULATION DATA

As a proof of concept, we applied our procedure to a collection of individuals, the ALSPAC data set (Boyd et al., 2013; Fraser et al., 2013) of 1,927 population individuals who had been whole‐genome sequenced (ascertainment and study numbers are provided in Supporting Information). The ALSPAC study website contains details of all the data that are available through a fully searchable data dictionary and variable search tool (http://www.bristol.ac.uk/alspac/researchers/our-data/).

To keep the total number of variants (SNPs) to a manageable level we focused on the 1,180,279 variants on chromosome 17 and, specifically, on the BRCA1 gene on this chromosome, but this number of variants is still comparable to that obtained in whole‐exome sequencing analysis. We removed monomorphic variants, which left 890,546 of the 1,180,278 variants. Then we removed common variants with minor allele frequencies >0.05, a common filtering practice (Rauch et al., 2015), which left 668,060 variants. The *Clinvar* database (https://www.ncbi.nlm.nih.gov/clinvar/?term=BRCA1[gene]) listed 2,563 pathogenic variants in the BRCA1 gene, of which 2,563 variants had a known dbSNP identifier. Of these variants, 14 occurred in the 1,927 ALSPAC individuals and, of the 14, 11 were polymorphic. One of these pathogenic variants had been removed owing to a high allele frequency so that 10 rare pathogenic BRCA1 variants remained (Table S1). No individual was homozygous for the minor allele at any of these 10 rare variants, and 61 individuals were heterozygous at one of these variants but never at more than one of these variants.

Initially, we carried out a standard case‐control association analysis assuming dominance of the minor allele for each variant. The 61 carriers of rare pathogenic variants were taken to be affected while the remaining 1,866 individuals were considered unaffected. This analysis was carried out with the *plink* program version 1.9 (Chang et al., 2015). Results (Tables S1 and S2) demonstrate that only two of the 10 rare variants were significantly detected by *plink*. This relatively low success rate reflects the extreme heterogeneity of the pathogenic variants.

For our SGS analysis, we worked only with the 61 ALSPAC carriers of pathogenic BRCA1 variants while the case‐control analysis referred to above also considered 1,866 control individuals. We removed the two variants easily detected by *plink* and proceeded with the remaining 668,058 chromosomes 17 variants and their genotypes in the 61 individuals. The first step was to run the *PH* program for each of the 61 carriers, that is, to generate segments of increasing and decreasing *H* values throughout chromosome 17, with each segment containing a maximum *H* value, *H*_max_. Each individual furnished from 9 to 17 *H*_max_ values and associated genomic positions for a total of 687 (average of 11.3) segments and *H*_max_ values in all 61 individuals. We designated a variant in the middle of the BRCA1 gene to represent the gene. It turned out that one of the segments in each of the 61 carriers contained this BRCA1 variant.

The segments of variants surrounding *H*_max_ values tended to be rather wide. The average width overall of 687 segments were 6.8 MB. Considering only those 61 segments containing that BRCA1 variant, the average segment width was 8.0 MB. Also, there was considerable overlap among segments. When the 687 segments were ordered by their start positions, any two consecutive segments exhibited some degree of overlap. That is, the segments appeared to be “smeared” over the length of the whole chromosome. Thus, we did not make further use of these segments and focused on the 687 *H*_max_ values and their positions and considered these as candidate disease variant positions.

We devised a shared genomic segment analysis as follows. For each variant, we determined whether any of the 687candidate disease variant positions were within ±*d* kb of the given variant position. We tried values of *d* = 2, 5, 10, 20, 50, 100, 200, and 500 kb. For each *d* value, we determined the empirical significance level that *N*_f_ or more individuals shared the given variant (i.e., were within *d* kb of the variant position). Under our null hypothesis, variant positions can be anywhere in the genome, so for each variant separately, we randomly permuted their genomic positions (Manly, 2007), which was performed on the basis of 10,000 permutation samples, including the observed data, so that the smallest possible significance level of 0.0001 should be interpreted as <0.0001. This step was carried out with our *shared SNP* program (http://lab.rockefeller.edu/ott/programs). As Table 1 shows, significance levels depend much on the distance *d* from a given DNA variant, within which *H*_max_ positions are captured. For example, for *d* = 100 kb, the occurrence of *H*_max_ positions in *N*_f_ = 5 or more individuals is significant with *p* = .0338 while fewer than five *H*_max_ positions occurring within ±100 kb may well happen by chance (*p* > .0995). Clearly, there is a tradeoff—more individuals can be captured with increasing *d*, but there is also an increased number *N*_f_ of individuals necessary for significance.

**Table 1:** Significance levels associated with numbers *N*_f_ of individuals within a distance *d* from a given variant

		Significance level for values of *d*					
*N*_f_	5	10	20	50	100	200	500
1	0.0907	0.1695	0.3093	0.5956	0.8296	0.9709	0.9999
2	**0.0049**	**0.0152**	0.0516	0.2279	0.5209	0.8604	0.9987
3	0.0001	0.0009	**0.0049**	0.0606	0.2548	0.6679	0.9924
4	0.0001	0.0001	0.0006	**0.0128**	0.0995	0.4484	0.9735
5	0.0001	0.0001	0.0001	0.0024	**0.0338**	0.2583	0.9296
6	0.0001	0.0001	0.0001	0.0004	0.0099	0.1333	0.8475
7	0.0001	0.0001	0.0001	0.0001	0.0031	0.0568	0.7308
8	0.0001	0.0001	0.0001	0.0001	0.0006	**0.0238**	0.5897
9	0.0001	0.0001	0.0001	0.0001	0.0002	0.0088	0.4360
10	0.0001	0.0001	0.0001	0.0001	0.0001	0.0021	0.2990
11	0.0001	0.0001	0.0001	0.0001	0.0001	0.0007	0.1903
12	0.0001	0.0001	0.0001	0.0001	0.0001	0.0001	0.1101
13	0.0001	0.0001	0.0001	0.0001	0.0001	0.0001	0.0624
14	0.0001	0.0001	0.0001	0.0001	0.0001	0.0001	**0.0310**
15	0.0001	0.0001	0.0001	0.0001	0.0001	0.0001	0.0155
16	0.0001	0.0001	0.0001	0.0001	0.0001	0.0001	0.0064

*Note*: The bold values are the largest value of *p*  < .05 for each value of *d*.

Of the total of 668,058 variants on chromosome 17, only some of them were shared by two or more individuals within a distance *d* of the variants’ positions. Clearly, the number of such “successful” variants very much depends on the value of *d* (right‐most column of Table 2).

**Table 2:** Number of runs, *N*_r_, of variants capturing a significant (*p* < .05) number, *N* > *N*_crit_, of individuals, where *H*_max_ positions within ±*d* kb of a given variant are considered

*d*	*N*_r_	*N*_crit_	*L*_avg_	*L*_1_	*L*_2_	*N*_var_
2	7	3	0.076	–	–	217
5	14	3	0.043	0.005	–	940
10	24	3	0.033	0.015	–	2,845
20	50	3	0.022	0.035	–	10,443
50	46	4	0.044	0.021	–	19,075
100	59	5	0.067	0.017	0.039	37,327
200	58	8	0.120	–	–	34,236
500	17	14	0.275	–	–	44,447

*Note: L*_avg_, average length of runs in kb. *L*_1_ and *L*_2_ are length(s) of runs overlapping the BRCA1 area (there was usually only one such run). *N*_var_,  number of variants capturing a number of individuals, *N* > *N*_crit_, *N*_crit_ is the smallest number of families captured with *p*  < .05, but we set a minimum, *N*_crit_ > 3.

**Figure 1:**
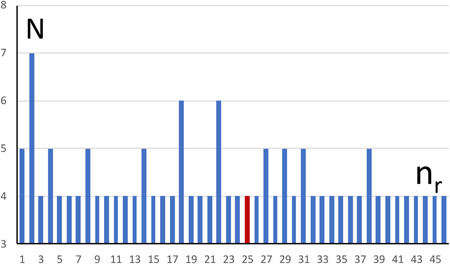
The graph shows the 46 runs of variants shared by *N* = 4 or more individuals (*y*‐axis) within *d* = 50 kb of a variant (see Table 2). Runs are numbered consecutively, *n*_r_ (*x* axis), with *N* = number of individuals (*y*‐axis) in a given run. The red bar (run 25) represents a run containing a variant within the BRCA1 area

An important question was now, which variants are shared by a *significant* number *N*_f_ of individuals? For example, for *d* = 100 kb, we found that 37,327 variants are shared by 5 or more individuals (Table 2, right‐most column), which represents a considerable reduction from the total of 668,058 variants we started out with. As many variants were within a short distance of each other, we compiled the number of runs of variants shared by significant numbers of individuals. For example, for *d* = 50, we found 46 runs of variants shared by four or more individuals (Table 2). This result is graphically shown in Figure 1, where we defined a BRCA1 area as the DNA region comprising the BRCA1 gene with an additional 1 MB extending at either end of the gene. For *d* < 5 kb, no variants shared by three or more individuals were in the BRCA1 region.

To summarize results for BRCA1, we started out with 668,058 variants on chromosome 17. Focusing on those variants shared by a significant number of individuals, this number was considerably reduced, for example, to 19,075 for a segment of width 2*d* = 100 kb surrounding variants shared by four or more individuals (Table 2). A further reduction was possible by focusing on runs of variants shared by 4 or more individuals; for *d* = 50 kb, there are only 46 such runs, with one of them in the BRCA1 region (Figure 1). Thus, a suitable strategy appears to be as follows: (a) For each individual or family, obtain a number of candidate variant positions (*H*_max_ values) with the *PH* program, possibly after deleting variants that are monomorphic or too common, but any approaches may be used at this step as long as they provide candidate variants for individuals. (b) Run the *shared SNP* program to identify variants shared by a significant number of individuals, where the relevant *d* value is not too large but large enough so that two or more individuals will share multiple variants (Table 2). The exact *d* value does not seem to be crucial but smaller *d* values will furnish fewer candidate runs of *N*_f_. (c) Run the *sigruns* program to find runs of variants shared by significant numbers of individuals. These programs have been written in Pascal and are available at http://lab.rockefeller.edu/ott/programs for Windows and Linux.

At Step 1 above, one might consider using only the highest‐ranked test statistics (here, *H*_max_ values). In our experience with the BRCA1 data, the candidate variants closest to the BRCA1 gene were never ranked highest and often were in the bottom half of ranks within one individual. Thus, using only top‐ranked candidate variants in each individual will often miss the most important variants.

## 5 DISCUSSION

As noted above, null hypothesis situations are created by permuting variant genotypes over genomic locations for one variant after another. Clearly, this destroys any linkage disequilibrium (LD) structure. However, our approach does not appeal to LD and is designed to work with one variant at a time. In this sense, the effects of LD are not relevant to our approach.

A particular advantage of SGS analysis is that test statistics (like *H*_max_ values) do not need to be “strong” in the sense that we are not using the values but only their locations. It is the sharing of a segment of positions that drives this analysis, whatever the individual shared values are. Conceivably, sizes of test statistics could somehow be incorporated as weights in SGS analysis, but we have not pursued this further as our current approach appears highly promising.

The method developed here provides a list of candidate genomic regions that are expected to harbor disease susceptibility variants. In this sense, it may be used as an aid to support other analyses, for example, linkage analyses of family data. Here, we applied a specific approach to find candidate variants for a dominant disease, but any method that generates candidate disease variants would be suitable for our SGS methodology. However, the degree, extent, and pattern of sharing may depend on penetrances and allele frequencies, so the performance of our SGS approach may vary depending on these factors.

## 6 SOFTWARE

Software (*sharedSNP* program, *sigruns* program) developed for our SGS analysis is freely available at http://lab.rockefeller.edu/ott/programs. Here, these programs have used output from the *PH* program, which is available from the same website.

Software package PLINK version 1.9; authors: Shaun Purcell, Christopher Chang. URL: www.cog-genomics.org/plink/1.9/.

## CONFLICT OF INTERESTS

The authors declare that there are no conflict of interests.

## ETHICS STATEMENT

Ethical approval for the study was obtained from the ALSPAC Ethics and Law Committee and the Local Research Ethics Committees.

## Supporting information

Supporting information

Supporting information

## Data Availability

The data that support the findings of this study are available from the ALSPAC resource at http://www.bristol.ac.uk/alspac/researchers/access/.
