# Supplementary material for: Shared genomic segment analysis with equivalence testing
Source: Genet Epidemiol. 2020 Jul 16;44(7):741–7. doi: 10.1002/gepi.22335 (PMC7540579; doi:10.1002/gepi.22335)
Supplement: Supplementary file 1 — Supporting information [file GEPI-44-741-s001.docx]

**Shared genomic segment analysis with equivalence testing**

Sukanya Horpaopan, Cathy S. J. Fann, Mark Lathrop, and Jurg Ott

# Supporting Information

# ALSPAC study numbers

Pregnant women resident in Avon, UK with expected dates of delivery 1st April 1991 to 31st December 1992 were invited to take part in the study. The initial number of pregnancies enrolled is 14,541 (for these at least one questionnaire has been returned or a “Children in Focus” clinic had been attended by 19/07/99). Of these initial pregnancies, there was a total of 14,676 foetuses, resulting in 14,062 live births and 13,988 children who were alive at 1 year of age.

When the oldest children were approximately 7 years of age, an attempt was made to bolster the initial sample with eligible cases who had failed to join the study originally. As a result, when considering variables collected from the age of seven onwards (and potentially abstracted from obstetric notes) there are data available for more than the 14,541 pregnancies mentioned above.

The number of new pregnancies not in the initial sample (known as Phase I enrolment) that are currently represented on the built files and reflecting enrolment status at the age of 18 is 706 (452 and 254 recruited during Phases II and III respectively), resulting in an additional 713 children being enrolled. The total sample size for analyses using any data collected after the age of seven is therefore 15,247 pregnancies, resulting in 15,458 foetuses. Of this total sample of 15,458 foetuses, 14,775 were live births and 14,701 were alive at 1 year of age.

A 10% sample of the ALSPAC cohort, known as the Children in Focus (CiF) group, attended clinics at the University of Bristol at various time intervals between 4 to 61 months of age. The CiF group were chosen at random from the last 6 months of ALSPAC births (1432 families attended at least one clinic). Excluded were those mothers who had moved out of the area or were lost to follow-up, and those partaking in another study of infant development in Avon.

# Rare pathogenic BRCA1 variants in the ALSPAC dataset

| **ch** | **SNP** | **bp** | ***maf*** | **Rank in *plink* analysis** |
| --- | --- | --- | --- | --- |
| 17 | rs56195342;rs80357895 | 41,215,385 | 0.00026 | 6,202 |
| 17 | rs1800744;rs80357534 | 41,226,488 | 0.00441 | 10 |
| 17 | rs80358340;rs28897689 | 41,243,509 | 0.00882 | 1 |
| 17 | rs80357407;rs80357711 | 41,243,512 | 0.00026 | 6,202 |
| 17 | rs80357666;rs28897687 | 41,243,840 | 0.00052 | 95 |
| 17 | rs80356923 | 41,243,908 | 0.00026 | 6,202 |
| 17 | rs80357631;rs28897683;rs80357830 | 41,245,071 | 0.00052 | 95 |
| 17 | rs80357467;rs80357957 | 41,245,233 | 0.00026 | 6,202 |
| 17 | rs55650082 | 41,245,759 | 0.00026 | 6,202 |
| 17 | rs199522616 | 41,258,509 | 0.00026 | 6,202 |
|  | Total |  | 0.00158 |  |

Table S1. Rare pathogenic BRCA1 variants, minor allele frequency *maf* < 0.01. Six of the ten variants showed a single minor allele, none of the variants were homozygous for the minor allele, and each variant had 1,927 genotypes. For confidentiality reasons, detailed genotype numbers cannot be exhibited.

| **ch** | **SNP** | **p_2_** |  | **SNP** | **p_2_** |
| --- | --- | --- | --- | --- | --- |
| 17 | rs192640538 | 0.0001 |  | rs188712267 | 0.0001 |
| 17 | rs191693441 | 0.0001 |  | rs189290149 | 0.0001 |
| 17 | rs147852474 | 0.0001 |  | rs111695603 | 0.0001 |
| 17 | . | 0.0001 |  | rs113855959 | 0.0001 |
| 17 | rs187509786 | 0.0001 |  | . | 0.0008 |
| 17 | . | 0.0001 |  | . | 0.0008 |
| 17 | rs112359384 | 0.0001 |  | rs186396362 | 0.0011 |
| 17 | rs111299379 | 0.0001 |  | . | 0.0011 |
| 17 | . | 0.0001 |  | rs138475796 | 0.0026 |
| 17 | **rs1800744;rs80357534** | 0.0001 |  | . | 0.0026 |
| 17 | **rs80358340;rs28897689** | 0.0001 |  | . | 0.0159 |
| 17 | . | 0.0001 |  | . | 0.0159 |
| 17 | rs189017742 | 0.0001 |  | . | 0.0159 |
| 17 | rs188039258 | 0.0001 |  | rs138533718 | 0.0162 |
| 17 | rs187396718 | 0.0001 |  | rs113789786 | 0.0162 |
| 17 | rs189070729 | 0.0001 |  | rs113895080 | 0.0207 |

Table S2. Results of dominant case-control association analysis by *plink* program. Shown here are the 32 variants with *p*_2_ < 0.05, where *p*_2_ = empirical significance level, adjusted for multiple testing (668,060 variants), obtained in 10,000 permutations of case-control labels (61 cases and 1,866 controls). The two bolded variants are in the BRCA1 gene (Table S1).
