# Supplementary material for: Shared genomic segment analysis with equivalence testing
Source: Genet Epidemiol. 2020 Jul 16;44(7):741–7. doi: 10.1002/gepi.22335 (PMC7540579; doi:10.1002/gepi.22335)
Supplement: Supplementary file 2 — Supporting information [file GEPI-44-741-s002.docx]

# Online Supporting Information

This supplement provides a detailed description of the ALSPAC data. Supporting Tables S1 and S2 list the rare pathogenic BRCA1 variants found in the ALSPAC data on chromosome 17. As requested by the ALSPAC ethics committee, detailed numbers of rare genotypes are not shown.
